# Sp1 Mediates a Therapeutic Role of MiR-7a/b in Angiotensin II-Induced Cardiac Fibrosis via Mechanism Involving the TGF-β and MAPKs Pathways in Cardiac Fibroblasts

**DOI:** 10.1371/journal.pone.0125513

**Published:** 2015-04-29

**Authors:** Rui Li, Jie Xiao, Xiaoteng Qing, Junhui Xing, Yanfei Xia, Jia Qi, Xiaojun Liu, Sen Zhang, Xi Sheng, Xinyu Zhang, Xiaoping Ji

**Affiliations:** 1 The Key Laboratory of Cardiovascular Remodeling and Function Research, Chinese Ministry of Education and Chinese Ministry of Public Health, Qilu Hospital of Shandong University, Jinan, Shandong, China; 2 Department of Emergency, Qilu Hospital, Shandong University, Jinan, Shandong, China; 3 Department of Cardiology, Central Hospital of Zibo, Shandong, China; 4 Department of Cardiology, Qilu Hospital of Shandong University, Qingdao, Shandong, China; University of Louisville, UNITED STATES

## Abstract

MicroRNA-7a/b (miR-7a/b) protects cardiac myocytes from apoptosis during ischemia/reperfusion injury; however, its role in angiotensin II (ANG II)-stimulated cardiac fibroblasts (CFs) remains unknown. Therefore, the present study investigated the anti-fibrotic mechanism of miR-7a/b in ANG II-treated CFs. ANG II stimulated the expression of specific protein 1 (Sp1) and collagen I in a dose- and time-dependent manner, and the overexpression of miR-7a/b significantly down-regulated the expression of Sp1 and collagen I stimulated by ANG II (100 nM) for 24 h. miR-7a/b overexpression effectively inhibited MMP-2 expression/activity and MMP-9 expression, as well as CF proliferation and migration. In addition, miR-7a/b also repressed the activation of TGF-β, ERK, JNK and p38 by ANG II. The inhibition of Sp1 binding activity by mithramycin prevented collagen I overproduction; however, miR-7a/b down-regulation reversed this effect. Further studies revealed that Sp1 also mediated miR-7a/b-regulated MMP expression and CF migration, as well as TGF-β and ERK activation. In conclusion, miR-7a/b has an anti-fibrotic role in ANG II-treated CFs that is mediated by Sp1 mechanism involving the TGF-β and MAPKs pathways.

## Introduction

Cardiac fibrosis involves the excessive accumulation of extracellular matrix (ECM) in the heart, which leads to cardiac dysfunction, and is closely associated with numerous cardiovascular diseases, including hypertension, myocardial infarction and cardiomyopathy. As the most common cell type in the heart, cardiac fibroblasts (CFs) play a pivotal role in the development of cardiac fibrosis via the excessive synthesis of collagens and the degradation of ECM via the production of matrix metalloproteinases (MMPs). The renin-angiotensin system (RAS), particularly angiotensin II (ANG II), is considered to be profoundly involved in the pathogenesis of cardiac fibrosis [1, 2] and plays a crucial role in cardiac remodeling. ANG IIincreases collagen expression, proliferation and migration in CFs by activating a variety of cell signaling pathways such as transforming growth factor β (TGF-β) and mitogen-activated protein kinases (MAPKs) pathways, which promote the differentiation, proliferation and migration of CFs [3–6].

Specific protein 1 (Sp1), which is a ubiquitously expressed transcription factor, is implicated in the regulation of several genes, including housekeeping genes and actively regulated genes, primarily via the involvement of their basal promoter activity. Growing evidence has demonstrated that Sp1 plays an important regulatory role in the expression of several genes relevant to fibrosis, including collagen I, TGF-β and downstream targets of TGF-β, such as MMPs [7–11]. Several studies have emphasized the significance of Sp1 in modulating the expression and deposition of collagen I under fibrotic conditions [12–16], and the capability of ANG II in stimulating Sp1 activation in adult CFs and in mouse hearts [10, 17, 18]. However, clear evidence of Sp1 regulation and its role in regulating collagen I production in ANG II-stimulated neonatal CFs remains lacking.

MicroRNAs (miRNAs, miRs) represent a class of naturally occurring endogenous small noncoding RNA molecules that are distinct from but related to siRNAs and that regulate their targets by inhibiting translation and/or by promoting mRNA degradation [19]. Increasing evidence has demonstrated that miRs are key regulators of genes involved in the pathophysiology of fibrosis in the heart [20–26]. miR-133 and miR-30 decrease the expression of connective tissue growth factor (CTGF) [20], and miR-21 contributes to cardiac fibrosis by enhancing ERK phosphorylation and increasing MMP-2 activity [22, 23]. As miR deregulation in the later stages of cardiac remodeling most likely functions as a compensatory mechanism and miR-7a was down-regulated in rats 5 days after transverse aortic constriction surgery, after which its expression returned to normal levels 20 days later [27], we therefore set out to investigate whether miR-7a/b is involved in cardiac fibrosis.

Taken together, because Sp1 regulates the synthesis of collagen I, and because collagen I is a predicted target of rat miR-7a/b, Sp1 may also function in the regulation of collagen I by miR-7a/b in neonatal CFs. Therefore, the purpose of this study was to experimentally identify the effect of Sp1 on the anti-fibrotic role of miR-7a/b in neonatal CFs, thereby presenting a viable target for therapeutic intervention of fibrotic cardiovascular diseases.

## Materials and Methods

### Ethics statement

This study complied with the Animal Management Rules of the Ministry of Public Health, People’s Republic of China (document No. 55, 2001), and the experimental protocol was approved by the Animal Ethics Committee of Qilu Hospital, Shandong University. All efforts were made to minimize suffering.

### Cell cultures and treatments

Wistar rats (3 days old) were purchased from the Laboratory Animal Services Centre (College of Medicine, Shandong University). Primary CFs were obtained by outgrowth from the left ventricles as previously described [28]. Briefly, hearts from 3-day-old rats were finely minced and mechanically digested with type II collagenase (120 units/mL; Sigma) by a rotor in a flask. The dispersed cells were placed in a culture flask for 90 min at 37°C in a CO_2_ incubator to separate the fibroblasts and cardiomyocytes. The fibroblasts were cultured in high-glucose Dulbecco’s modified Eagle medium (DMEM) supplemented with 10% newborn calf serum, 5% fetal bovine serum, penicillin (100 U/ml), and streptomycin (100 mg/ml). CFs at passage 2 or 3 was used. Fibroblasts were checked for purity by staining for the fibroblast marker vimentin using an anti-vimentin antibody (Santa Cruz, CA) and were distinguished from myocytes and endothelial cells by staining for α-actin (Santa Cruz, CA) and CD31 (Abcam, Cambridge), respectively. Over 95% of the cultured cells was vimentin positive and α-actin and CD31 negative. Then, the cells were cultured for 24 h in serum starvation medium and pretreated with or without mithramycin 1 h before stimulation with ANG II (100 nM) for 24 h.

### miR transfection

The CFs were transiently transfected with miR-7a/b mimics or miR-7a/b inhibitors or their negative control siRNA (NC) (GenePharma, Shanghai, China) using Lipofectamine 2000 reagent (Invitrogen, Carlsbad, CA, USA) according to the manufacturer’s protocol, as described previously [29]. The sequences of the miR-7a/b mimics were designed as follows: 5′- UGGAAGACUAGUGAUUUUGUUGU-3′/5′- UGGAAGACUUGUGAUUUUGUUGU-3′. The sequences of the miR-7a/b inhibitors were designed as follows: 5′-ACAACAAAAUCACUAGUCUUCCA-3′/5′- ACAACAAAAUCACAAGUCUUCCA- 3′. The negative control siRNA was synthesized with the following sequence: 5′-UUCUCCGAACGUGUCACGUTT-3′.

### Western blot analysis

The preparation of whole cell lysates and Western blot analysis of protein expression were performed using routine procedures as described previously [29]. Primary antibodies were obtained from the following sources and used at 1:1000 dilution: anti-Sp1 (Millipore, Germany), anti-collagen I, anti-TGF-β, anti-matrix metalloproteinase-2 (anti-MMP-2), or anti-MMP-9 (Abcam, Cambridge), anti-p-ERK, anti-ERK, anti-p-JNK, anti-JNK, anti-p-p38, and anti-p38 (Cell Signaling Technology).

### Quantitative RT-PCR

miR-7a/b expression was evaluated using TaqMan Small RNA Assays (Applied Biosystems), and cDNA was synthesized using a TaqMan MicroRNA Reverse Transcription Kit (Applied Biosystems) with RT-U6 and miR-specific stem-loop primers according to the manufacturer’s instructions. miR-7a/b levels were measured using Taqman MicroRNA assays (Applied Biosystems) and Taqman Universal PCR Master Mix (Applied Biosystems). For Sp1 and collagen I mRNA quantification, total RNA was isolated using TRIzol reagent (Invitrogen), and cDNA synthesis was performed with oligo (dT) primers. Quantitative RT-PCR analysis was performed using a SYBR RT-PCR kit (Bio-Rad). The sequences of the primer pairs were as follows: Sp1, 5′-CCATCTTTGTCGATTGCTGA-3′ (sense) and 5′–AACGGTTTGATCTCCATCCC-3′ (antisense); collagen I, 5′-CCCTACCCAGCACCTTCAAA-3′ (sense) and 5′-CATGGAGATGCCAGATGGTTAG-3′ (antisense).

### Immunofluorescent staining and confocal microscopy

Briefly, CFs were seeded in 8-well Millicell EZ slides (Millipore, Germany). After treatment, the CFs were washed with cold phosphate buffered saline (PBS), and then fixed with 4% paraformaldehyde for 20 min at room temperature. After three washes with PBS, the CFs were blocked in 5% goat serum for 1 h and incubated overnight with primary antibodies for Sp1 (1:200, Cat. NO. sc-14027, Santa Cruz, CA, USA) and collagen I (1:500, Cat. NO. ab6308, Abcam, Cambridge) at 4°C. The next day, the CFs were washed with PBS before incubation with secondary antibodies, followed by incubation with DAPI. Images were captured in three blinded sessions by two blinded observers via laser scanning confocal microscopy (LSM710, Carl Zeiss, Germany) and analyzed with Image-Pro Plus 6.0 software.

### Luciferase assays

pRL-cmv vectors (Genechem, Shanghai) containing control siRNA or miR-7a/b mimics were co-transfected with wild type collagen I vector (with a 3′ UTR segment harboring the putative miR-7a/b binding sequence) or mutant collagen I vector (with a 3′ UTR segment harboring the putative miR-7a/b binding mutant sites). The CFs were harvested and lysed 24 h after transfection, and *Renilla* luciferase activities were measured consecutively using dual-luciferase assays (Promega), according to the manufacturer’s instructions.

### MTT assay

3-[4,5-Dimethylthiazol-2-yl]-2,5-diphenyl tetrazolium bromide (MTT, Sigma-Aldrich) was prepared as a 5mg/mL stock in PBS. The CFs were seeded in 96-well culture plates for the MTT assay, following the manufacturer’s instructions. Absorbance was measured at 540 nm to determine the percentage of cell survival.

### Cell migration assay

The CFs that were grown to confluence in 35 mm plates were transfected with miR-7a/b mimics, inhibitors, control siRNA or were treated with ANG II for 24 h. The monolayer-wounding cell migration assay was performed as previously described [28]. The results are expressed as the mean number of migrating cells per field. The cells were subsequently fixed and stained, and the percentage of polarized cells was calculated.

### Gelatin zymography

MMP enzyme activity was assayed in cell lysates by gelatin zymography. Briefly, samples were electrophoresed on 1 mg/mL gelatin by 10% SDS-PAGE; the gel was then renatured in washing buffer (50 mM Tris–HCl, 100 mM NaCl, and 2.5% Triton X-100), followed by a rinse in washing buffer without Triton X-100. After incubation in activation buffer (50 mM Tris–HCl, 150 mM NaCl, 10 mM CaCl_2_, 1 μM ZnCl_2_) at 37°C for 24 h, the gel was stained with Coomassie brilliant blue R-250 and then destained. Lytic bands from the gelatin digestion were indicative of MMP-2 (72 kDa) and MMP-9 (92 kDa) activity.

### Statistical analysis

The data are expressed as the means ± SD of three independent experiments. Differences between treatment groups were assessed with Student’s *t*-test and one-way ANOVA using SPSS 18.0 software (SPSS Inc., Chicago, IL, USA). p < 0.05 was considered statistically significant.

## Results

### Enhanced activation of Sp1 and collagen I in ANG II-treated CFs

**Fig 1:**
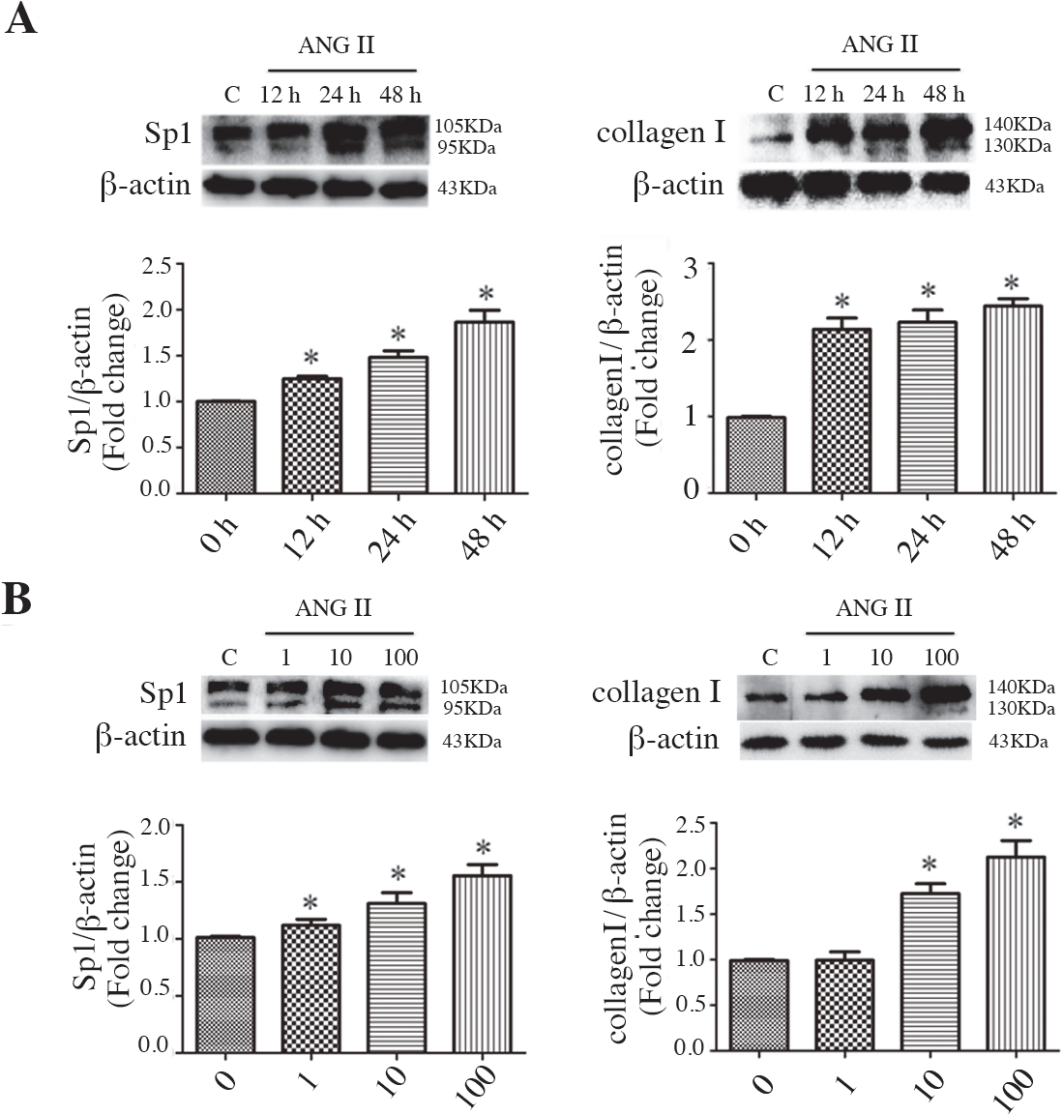
ANG II stimulated Sp1 and collagen I expression in CFs. A: Protein expression of Sp1 and collagen I in 100 nM ANG II-treated CFs at different time points. B: Protein expression of Sp1 and collagen I treated with different concentrations of ANG II for 24 h. C: normal untreated CFs. *p < 0.05, compared with control.

First, we examined the expression of Sp1 and collagen I in ANG II-treated CFs. The identification of CFs was presented as shown in S1 Fig ANG II (100 nM) effectively activated the expression of Sp1 and collagen I in a time-dependent manner (Fig 1A). Significant increases were detected as early as 12 h after the start of ANG II incubation, whereas a maximum effect was observed at 48 h. The dose response of ANG II was measured in the CFs at 24 h. The expression of Sp1 and collagen I increased gradually in a dose-dependent manner (Fig 1B). ANG II (100 nM) effectively increased Sp1 and collagen I expression at 24 h; therefore, this condition was used for subsequent experiments.

### miR-7a/b repressed ANG II-stimulated expression of Sp1 and collagen I in CFs

**Fig 2:**
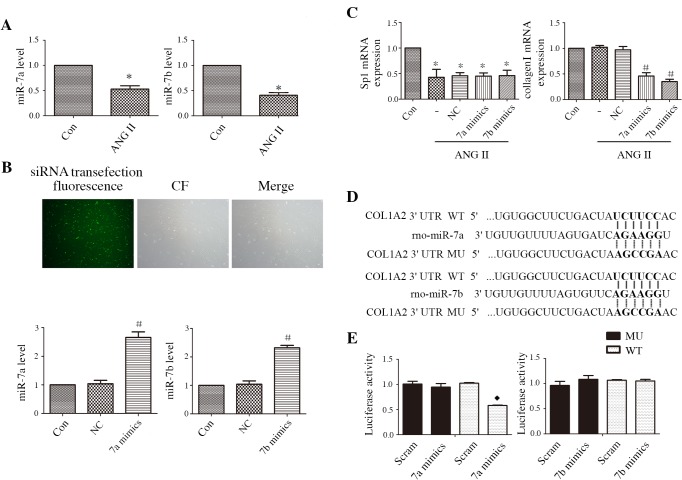
Effect of miR-7a/b on Sp1 and collagen I mRNA expression and luciferase assays results. A: The expression levels of miR-7a/b in normal untreated CFs and ANG II-treated CFs. B: Immunofluorescent staining of the transfection efficacy of siRNA in CFs and qRT-PCR analysis of the efficacy of miR-7a/b mimics. C: mRNA expression of Sp1 and collagen I in normal CFs and ANG II-treated CFs that were treated with NC siRNA or miR-7a/b mimics. D: Conserved miR-7a/b binding site in the 3′ untranslated region (UTR) of collagen I. E: Luciferase activity analysis after treatment with NC siRNA or miR-7a/b mimics. Con: untreated CFs;-: only ANG II-treated CFs. NC: negative control siRNA; Scram: Scrambled siRNA. WT: wild type of collagen I; MU: mutant type of collagen I. *p < 0.05, compared with control; #p < 0.05, compared with NC; and ◆p < 0.05, compared with NC-siRNA-WT.

To investigate whether miR-7a/b is involved in the expression of Sp1 and collagen I, we quantified miR-7a/b levels in ANG II-treated CFs using qRT-PCR. Compared with the levels in normal untreated CFs, miR-7a/b was down-regulated in CFs treated with ANG II (Fig 2A). Then, we transfected miR-7a/b mimics into CFs, with an efficacy of nearly 90%, and miR-7a mimics effectively up-regulated miR-7a expression and miR-7b mimics functionally up-regulated miR-7b expression (Fig 2B). Sp1 and collagen I mRNA expression was also evaluated. Sp1 mRNA expression decreased compared with normal untreated CFs, whereas collagen I mRNA expression remained unchanged under ANG II treatment. No significant differences were observed in ANG II-treated CFs that were pretreated with/without NC siRNA transfection; miR-7a/b mimics abrogated collagen I mRNA expression, but not the expression of Sp1 mRNA, upon ANG II treatment (Fig 2C).

Because collagen I is thought to be a miR-7a/b target (Fig 2D), we performed luciferase assays to confirm whether miR-7a/b directly targets collagen I. The results showed that transfecting with miR-7a mimics strongly inhibited luciferase activity, whereas no significant change was observed in CFs transfected with miR-7b mimics compared with CFs transfected with scrambled siRNA (Fig 2E).

**Fig 3:**
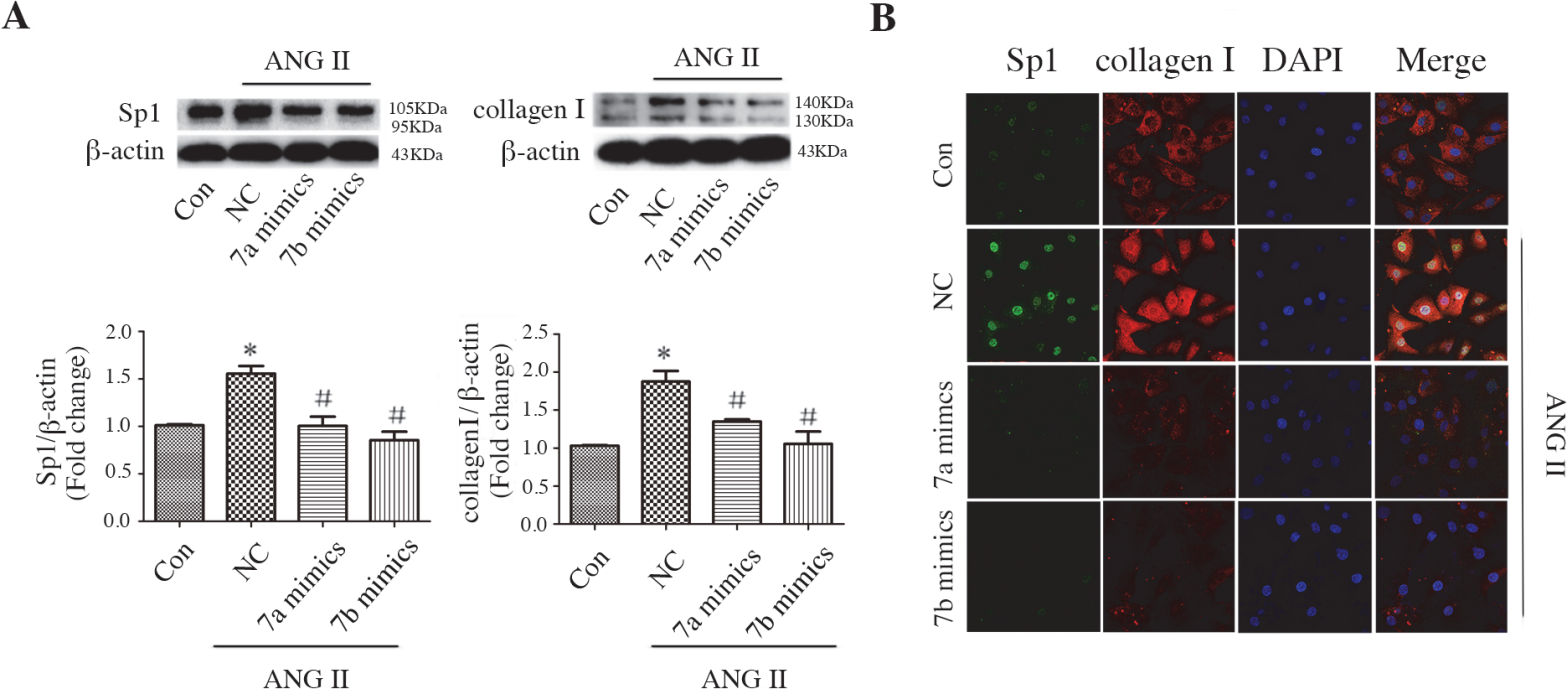
Effect of miR-7a/b mimics on the regulation of Sp1 and collagen Iexpression and localization in CFs. A: Western blots of Sp1 and collagen I in untreated CFs and ANG II-treated CFs that were treated with NC siRNA or miR-7a/b mimics. B: Immunofluorescent staining of Sp1 and collagen I location in normal untreated CFs and ANG II-treated CFs that were treated with NC siRNA or miR-7a/b mimics. Con: normal untreated CFs; NC: negative control siRNA. *p < 0.05, compared with control; and #p < 0.05, compared with NC.

We further quantified the protein expression and cellular localization of Sp1 and collagen I regulated by miR-7a/b in CFs. miR-7a/b repressed the elevated protein expression of Sp1 and collagen I induced by ANG II (Fig 3A). Immunofluorescence staining showed that Sp1 primarily localized to the nucleus in CFs and that its expression was closely correlated with collagen I expression when treated with ANG II with or without miR-7a/b mimics (Fig 3B).

### miR-7a/b inhibits CF proliferation and migration

**Fig 4:**
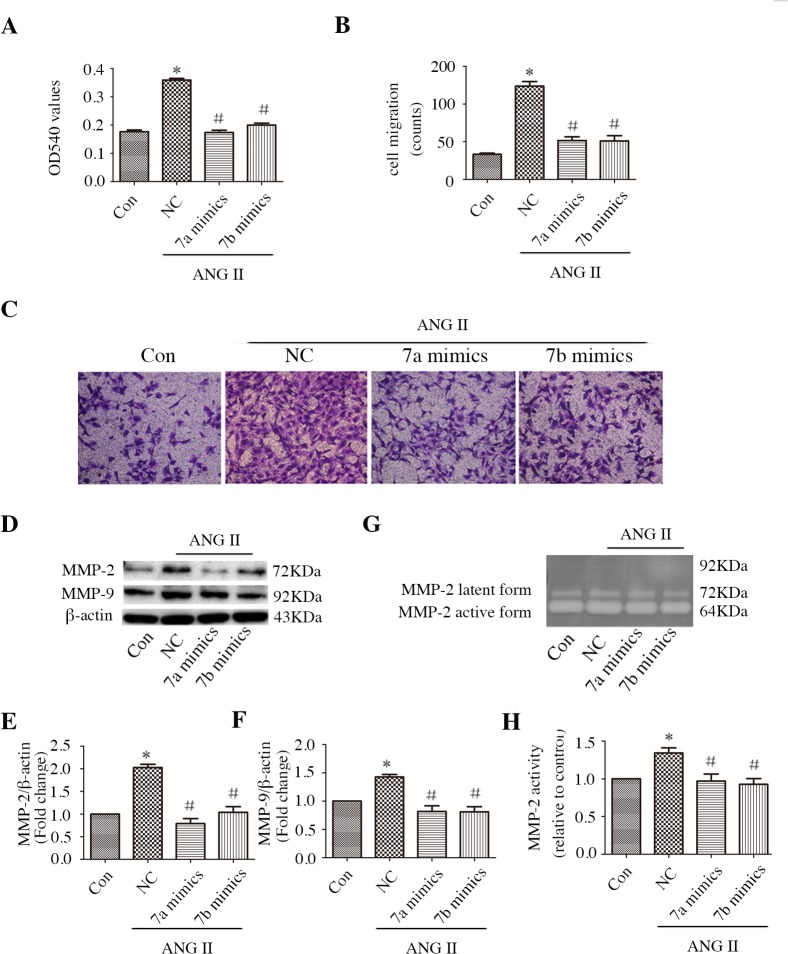
Effect of miR-7a/b mimics on proliferation, migration, and MMP-2 and MMP-9 expression/activity in CFs. A: MTT assay. B-C: CFs on the external surface of the Transwell were dyed with Crystal Violet and photographed under a microscope. D-F: Western blot analysis of MMP-2 (D, E) and MMP-9 (D, F) protein levels. G-H: Media were harvested for gelatin zymography analysis of MMP-2 activity; no MMP-9 activity was detected in the media. Con: normal untreated CFs; NC: negative control siRNA; *p, < 0.05 compared with control; and #p, < 0.05 compared with NC.

Because CF proliferation and migration play an important role in myocardial fibrosis, we investigated the proliferation of CFs using MTT assays. CFs displayed rapid growth over 24 h following ANG II stimulation, which was slowed by the miR-7a/b mimics (Fig 4A). In addition, the results of the Transwell assay demonstrated reduced migration by CFs treated with miR-7a/b mimics compared with CFs treated with control siRNA (Fig 4B and 4C). Then, we measured MMP-2 and MMP-9 expression and activity in ANG II-treated CFs. Compared with the control cells, miR-7a/b dramatically reduced the ANG II-induced protein expression of MMP-2 and MMP-9 (Fig 4D–4F), as well as the activity of MMP-2 (Fig 4G and 4H). MMP-9 activity was not detected.

### miR-7a/b reduced ANG II-induced TGF-β and MAPK activation

**Fig 5:**
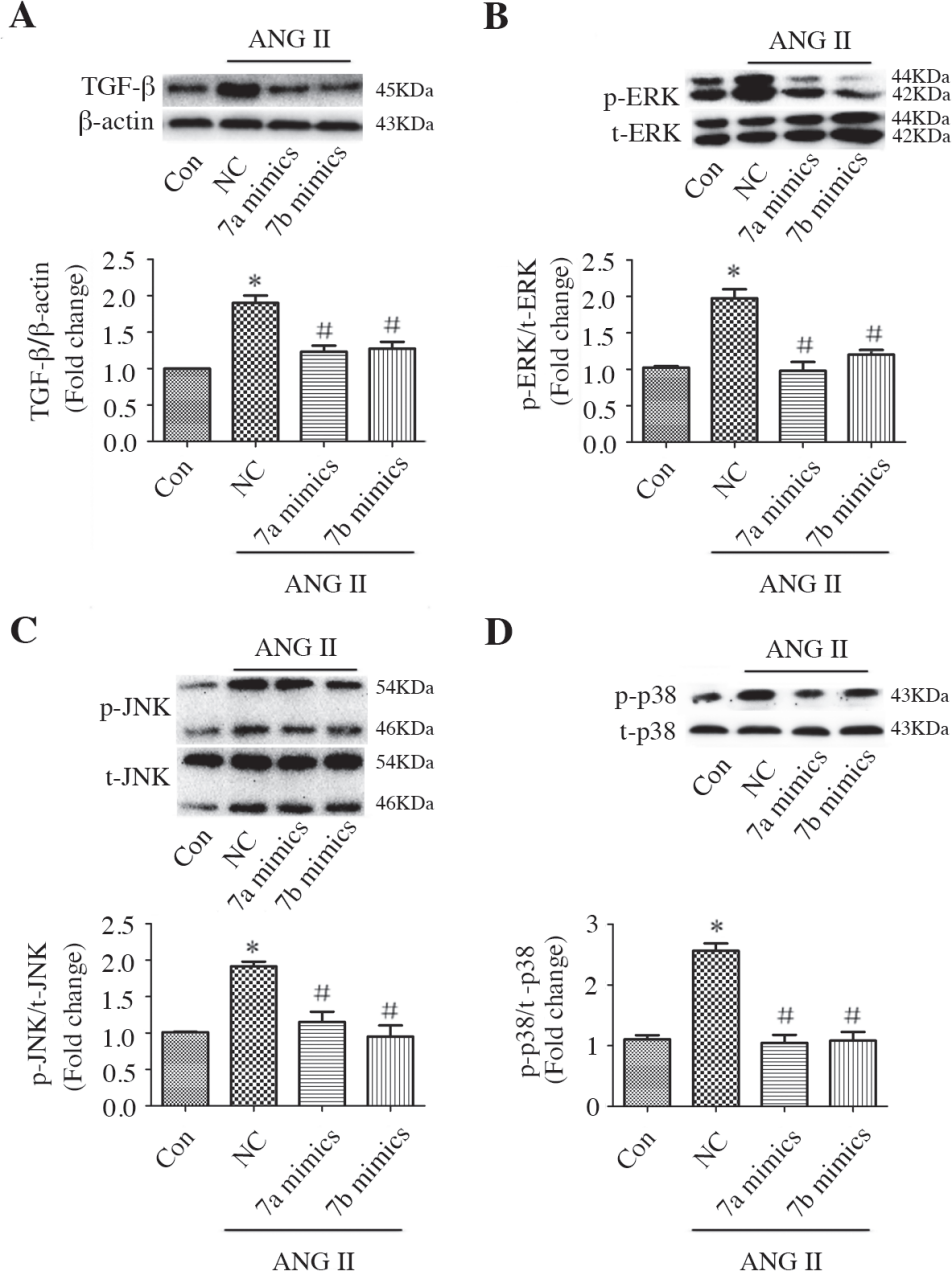
Effect of miR-7a/b mimics on TGF-β and MAPK pathways. Western blots and analysis of TGF-β (A), p-ERK (B), p-JNK (C), and p-p38 (D) expression. Con: normal untreated CFs; NC: negative control siRNA; *p < 0.05, compared with control; and #p, < 0.05 compared with NC.

To determine the potential signal transduction pathway involved in miR-7a/b-mediated fibrosis, we evaluated the TGF-β and MAPK pathways. TGF-β was activated by ANG II but was suppressed by miR-7a/b mimics (Fig 5A). Although miR-7a/b did not affect total ERK, JNK or p38 levels, miR-7a/b mimics effectively inhibited the ANG II-stimulated phosphorylation of ERK, JNK and p38 (Fig 5B–5D).

### Sp1 mediates miR-7a/b-regulated collagen Iexpression and TGF-β and ERK activation in CFs

**Fig 6:**
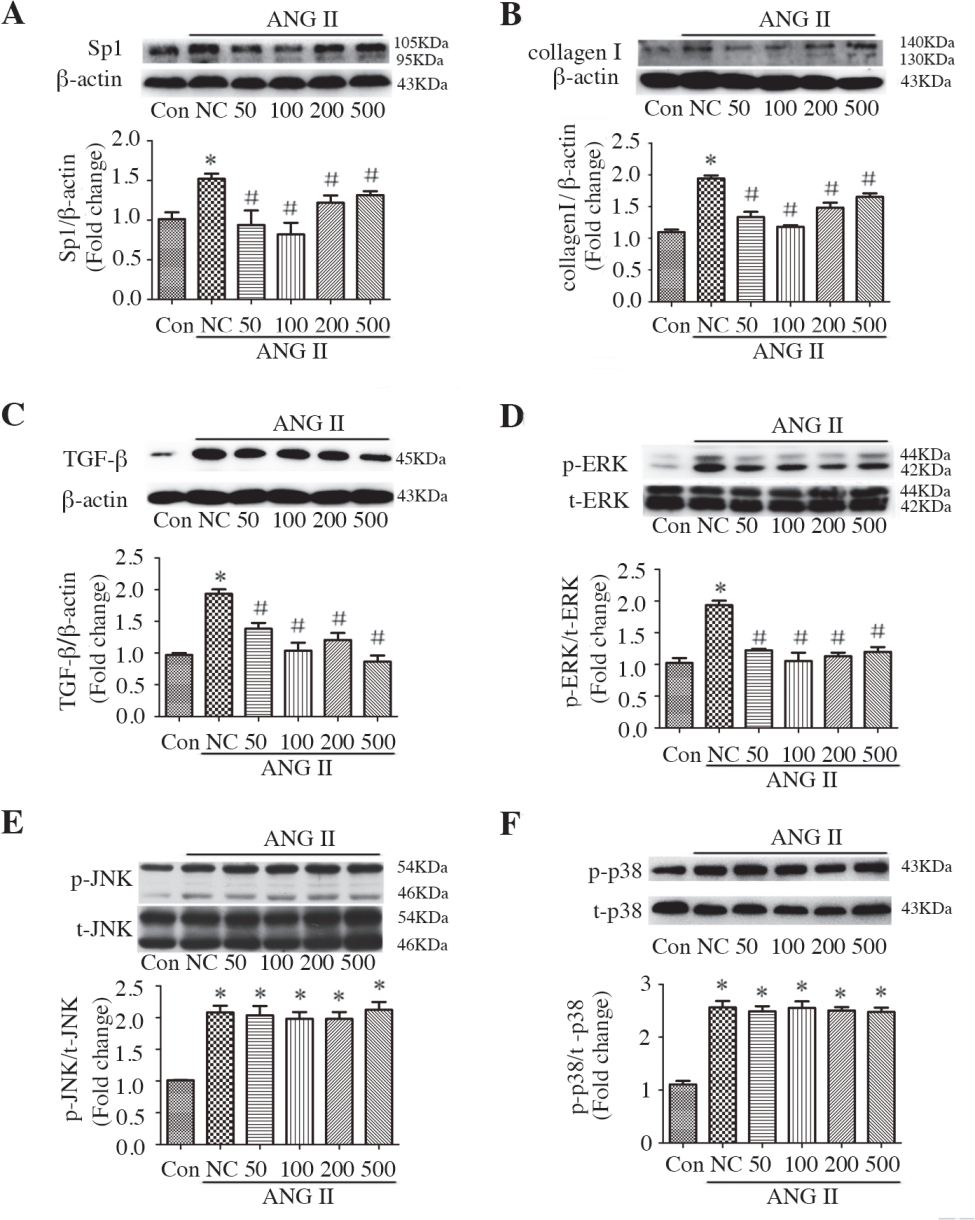
Effect of different concentrations of mithramycin on the regulation of Sp1, collagen Iexpression and signal transduction in CFs. A-F: Western blots and analysis of mithramycin on the regulation of Sp1 (A), collagen I (B), TGF-β (C), p-ERK (D), p-JNK (E) and p-p38 (F). Con: normal untreated CFs; NC: negative control siRNA; M: mithramycin. *p < 0.05, compared with control; and #p < 0.05, compared with NC.

**Fig 7:**
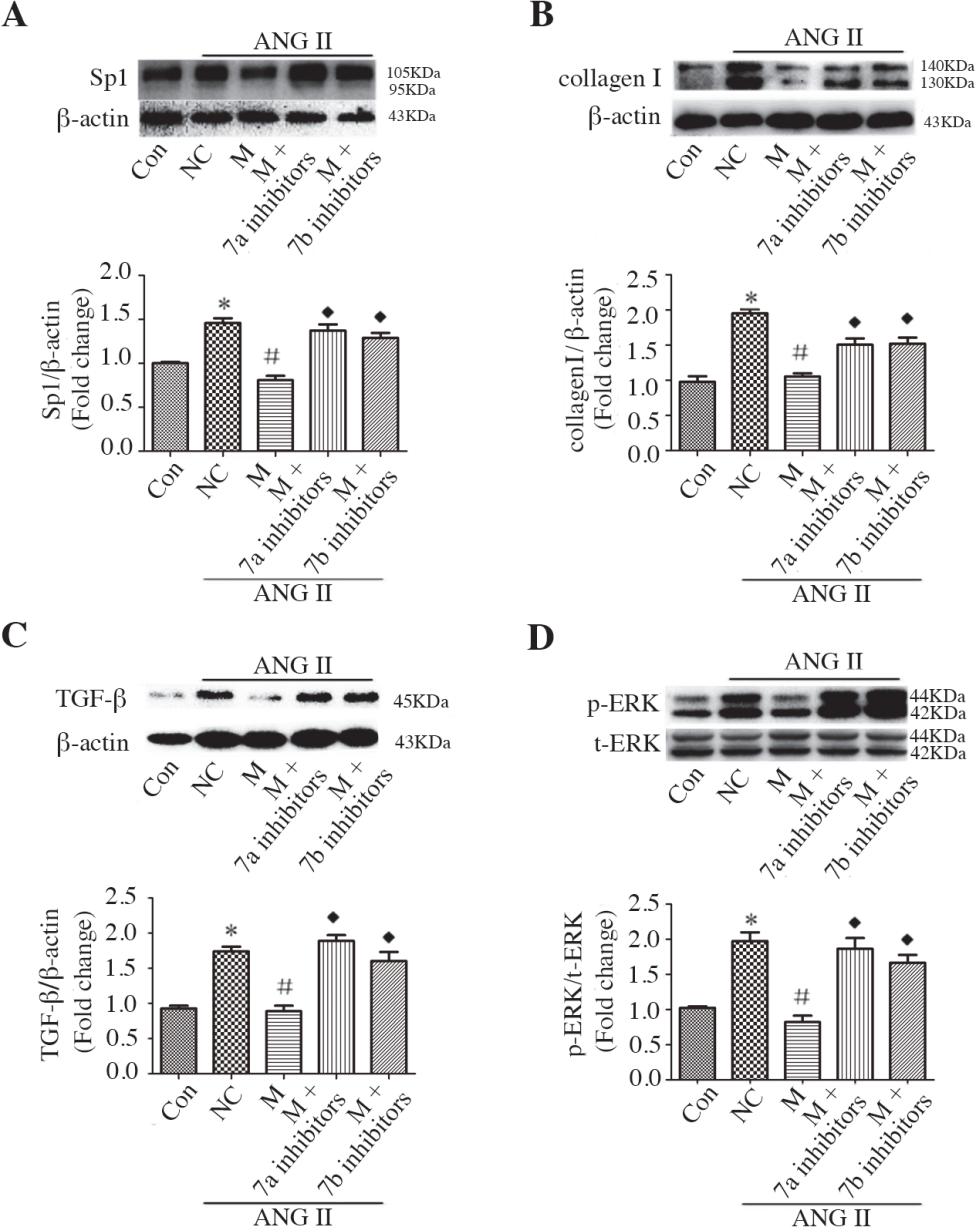
Sp1 mediates miR-7a/b-regulated collagen I expression and signal transduction in CFs. A-D: Western blots and analysis of Sp1-mediated miR-7a/b-regulated expression of Sp1 (A), collagen I (B), TGF-β (C) and p-ERK (D). Con: normal untreated CFs; NC: negative control siRNA; M: mithramycin. *p < 0.05, compared with control; and #p, < 0.05 compared with NC; and ◆p < 0.05, compared with mithramycin.

To characterize the potential role of Sp1 in miR-7a/b function, we investigated the effect of inhibiting Sp1 with mithramycin, with or without incubation with miR-7a/b inhibitors. The efficacy of miR-7a/b inhibition was evaluated by qRT-PCR, as shown in S2 Fig. Mithramycin attenuated ANG II-stimulated Sp1 and collagen I over-production (Fig 6A and 6B), as well as TGF-β and ERK activation (Fig 6C and 6D), in a dose-dependent manner; however, mithramycin did not affect JNK or p38 activation (Fig 6E and 6F). Subsequent experiments were performed using 100 nM mithramycin. Of note, the miR-7a/b inhibition reversed mithramycin-regulated Sp1 and collagen Iexpression (Fig 7A and 7B), and the inhibition of miR-7a/b amplified mithramycin-ameliorated TGF-β and ERK activation (Fig 7C and 7D).

### Sp1 mediates miR-7a/b-regulated MMPs and migration in CFs

**Fig 8:**
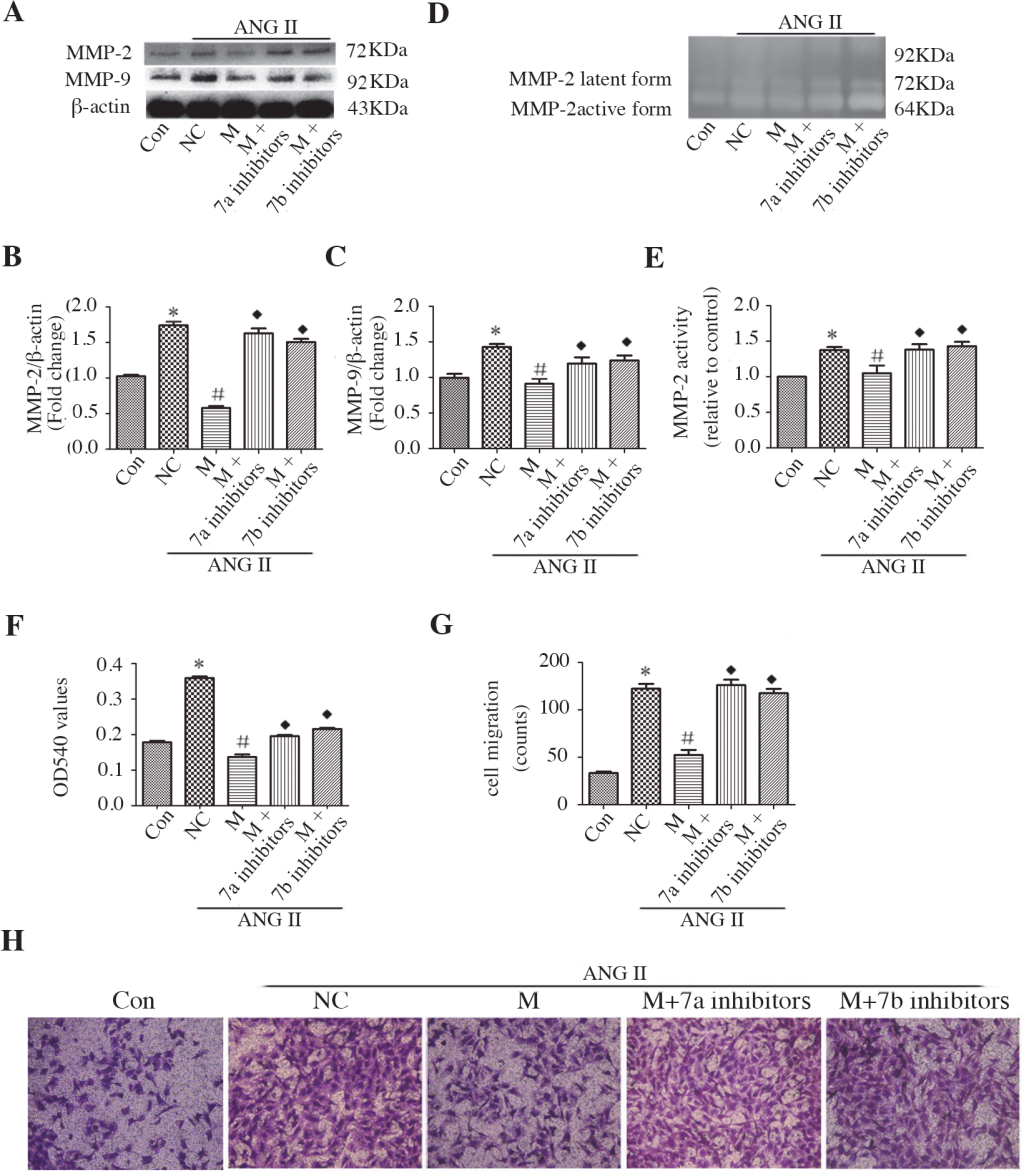
Sp1 mediates miR-7a/b-regulated proliferation migration, and MMP-2 and MMP-9 expression/activity in CFs. A-C: Western blot analysis of MMP-2 (A, B) and MMP-9 (A, C) protein levels. D-E: Media were harvested for gelatin zymography analysis of MMP-2 activity; no MMP-9 activity was detected in media. F: MTT assay. G-H: CFs on the external surface of the Transwell were dyed with Crystal Violet and photographed under a microscope. Con: normal untreated CFs; NC: negative control siRNA; M: mithramycin. *p < 0.05, compared with control; #p < 0.05, compared with NC; and ◆p < 0.05, compared with mithramycin.

To gain further insights into the potential mechanism by which Sp1 mediates miR-7a/b regulated fibrosis, we identified the involvement of Sp1 in the regulation of MMPs and migration in CFs. Mithramycin distinctly inhibited MMP-2 and MMP-9 expression (Fig 8A–8C), as well as MMP-2 activity (Fig 8D and 8E), whereas the miR-7a/b inhibitors abolished this effect. MTT assay showed that mithramycin attenuated CF proliferation, and the miR-7a/b inhibitors slightly offset this inhibitory effect (Fig 8F). Notably, miR-7a/b successfully reversed the effect of mithramycin on the number of migrated CFs (Fig 8G and 8H). Thus, the role of miR-7a/b in MMP expression and activity, as well as in CF migration, may be partially mediated by the inhibition of Sp1.

## Discussion

The present study was designed to investigate the molecular mechanism by which miR-7a/b antagonizes the ANG II-induced downstream proliferation and fibrosis signaling pathway in isolated CFs. We showed that 100 nM of ANG II promoted Sp1 and collagen I expression, as well as CF proliferation and migration. These changes were remarkably suppressed by the transfection of miR-7a/b mimics. Pretreatment with mithramycin blocked ANG II-stimulated Sp1 and collagen I expression; however, the miR-7a/b inhibitors counteracted the effect of mithramycin, which suggests that Sp1 mediates the regulation of collagen I by miR-7a/b. Similar results were obtained regarding the regulation of MMP expression and CF migration. Furthermore, miR-7a/b mimics inhibited the ANG II-stimulated activation of TGF-β, ERK, JNK and p38, and the regulation of TGF-β and ERK was also mediated by Sp1.

CFs, which are the predominant secretory cells that produce ECM proteins, are important targets of ANG II and play an irreplaceable role in the development of cardiac fibrosis, which results in cardiac dysfunction and the adverse outcome of heart failure. Because cardiac remodeling suggests that interfering with fibrosis may be of benefit to cardiac function [30–32], miRs have become increasingly crucial in regulating cardiac remodeling, based on their effects on fibrosis. miR-7, which is closely related to cell migration and proliferation [25, 26, 33, 34], appears to be of considerable importance in collagen synthesis based on the finding that both α1(I) and α2(I) collagens are predicted to be targets of miR-7 genes in dermal fibroblasts [26]. A major finding of the present study is that the ectopic expression of miR-7a/b was effective in suppressing collagen I at both the protein level and mRNA levels. Additionally, collagen I was identified as a novel target of miR-7a, but not of miR-7b, in CFs, which suggests that the effect of miR-7b on collagen I expression is not direct and that there may be other post-transcription mechanisms that contribute to this difference. However, it should be noted that we observed no alterations in collagen mRNA but an increase in collagen protein, which supports the finding that angiotensin II exert its effects on collagen mRNA abundance through indirect mechanisms and thereby alter collagen gene expression in serum-starved CFs [35].

In addition to miRs, many transcription factors are capable of modulating collagen I because collagen I contains four cis-response elements, which mediate the basal transcriptional activity of human collagen I; one of these cis-response elements is a repressor site, whereas the other three have been shown to be activator sites [36]. Among strong transcription factors, Sp1 is deeply involved in excessive expression and subsequent deposition of collagen I because Sp1 activates the human collagen I promoter via GC boxes and the TCCTCC motif, and its binding activity to collagen I promoter increases under fibrotic conditions [12, 37–39]. In the present study, we observed an increase in the protein expression of Sp1 in ANG II-treated neonatal CFs, which was consistent with findings that the levels of cardiac Sp1 increased in ANG II-induced hypertensive heart [10, 17] and in ANG II-treated adult fibroblasts [18]. However, Sp1 mRNA levels were down-regulated upon ANG IItreatment. Two mechanisms could account for this discrepancy. First, RNA is less stable than protein; therefore, other regulators may promote the degradation of Sp1 mRNA, such as miR-29b, which was shown to be up-regulated in ANG II-treated CFs [40] and directly targets Sp1 [41]. Second, posttranscriptional modifications such as phosphorylation, glycosylation, acetylation, sumoylation, and ubiquitination appeared to be capable of modulating Sp1 protein stability by increasing/decreasing degradation [42–44]. More experiments are needed to explore this hypothesis.

On the other hand, it is worth mentioning that miR-7a/b and mithramycin effectively suppressed the expression of Sp1 and collagen I in CFs, which may explain our unpublished observation that miR-7a/b strongly reduced the expression of Sp1 and collagen I in the border zones of hearts in a mouse model of myocardial infarction, suggesting that ANG II may be persistently overexpressed. In the present study, the finding that the inhibitory effect of mithramycin was reversed by miR-7a/b inhibition suggested that the interaction between miR-7a/b and collagen I was largely dependent on the presence of a certain level of Sp1 in the microenvironment. miR-7a can directly bind to the 3′UTR of Sp1 [45], which may be responsible for the Sp1-mediated miR-7a regulation of collagen I expression. No direct relationship between miR-7b and Sp1 was found.

Fibrosis is associated with the increased expression and activity of MMPs. MMP-2 stimulates collagen I expression in a time-dependent manner [46], and enhanced MMP activity is directly proportional to fibrosis, resulting in matrix remodeling and left ventricular (LV) enlargement [47]. In contrast, the inhibition of MMP activity hinders the migratory ability of CFs and prevents LV remodeling [48, 49]. Recent studies have demonstrated the critical role of Sp1 in regulating MMPs in CFs; namely, ANG II promotes CF migration by suppressing the MMP regulator reversion-inducing-cysteine-rich protein with Kazal motifs (RECK) via a mechanism dependent on Sp1 activation [18]. In addition, Sp1 knockdown reduced MMP-2 expression and activity in CFs [11]. Indeed, we found that ANG II stimulated MMP-2 expression/activation and MMP-9 expression in CFs, which were repressed by miR-7a/b mimics and mithramycin, suggesting that both miR-7a/b and mithramycin act as MMP repressors. Interestingly, MMP-9 activity was not detected in the culture medium, similar to findings reported for high-glucose stimulated medium in CFs [28], suggesting that MMP-9 has a less important role in ANG II-treated CFs than does MMP-2.

Sp1 also mediates collagen expression indirectly via the TGF-β signaling cascade because Sp1 drives the TGF-β activation of promoters, such as collagen α1(I) [50] and α2(I) chains [51]. The transcription of TGF-β is regulated by Sp1 binding to GC-rich sites in promoters of TGF-β [52]. Consistent with this concept, our present data indicated that Sp1 is essential in TGF-β up-regulation; based on the finding that mithramycin inhibited the expression of TGF-β. Sp1 regulation of TGF-β involvement in fibrosis was further supported by the findings that the up-regulation of the Sp1/ TGF-β pathway may be one mechanism, by which the deletion of Smad7 enhanced hypertensive cardiac remodeling, which suggests that Sp1 may be a crucial mediator of cardiac fibrosis and remodeling [10]. In addition to the TGF-β pathway, many *in vivo* and *in vitro* studies have demonstrated that ANG II activates intracellular MAPKs signaling cascades, which play a pivotal role in the development of cardiac fibrosis [5, 18, 53–55]. Mammalian MAPKs are grouped into three major subfamilies: ERK, which is always activated in response to cell growth, cell proliferation and differentiation, and JNK and p38, which are usually activated in response to inflammatory cytokines and cellular stress. We further demonstrated that the activation of p-ERK, p-JNK and p38 by ANG II was repressed by miR-7a/b mimics, which may contribute to the effects of miR-7a/b on collagen I expression and on CF proliferation and migration. However, Sp1 only mediated miR-7a/b-regulated TGF-β and ERK activation but not JNK or p38 activation, which suggests that there may by other mediators involved in miR-7a/b-regulated JNK and p38 activation.

## Conclusions

We presented data demonstrating that the overexpression of miR-7a/b reduced collagen I expression and suppressed both the TGF-β and MAPK pathways, which is an essential mechanism that may contribute to myocardial remodeling and fibrosis under chronic ANG II stimulation conditions. In addition, miR-7a/b inhibited CF proliferation and migration, thereby presenting a novel target for the therapeutic intervention of fibrotic heart diseases. Although our study revealed that Sp1 is a pivotal mediator of miR-7a/b activity in CFs, future research is required to determine the physiological effects of miR-7a/b and Sp1 in mice and humans.

## Supporting Information

S1 Fig Identification of CFs. Immunofluorescent staining of vemintin (first row), α-actin (second row) and CD31 (third row) in CFs.
(TIF)

S2 Fig qRT-PCR analysis of the efficacy of miR-7a/b inhibitors in CFs. Con: normal untreated CFs; NC: negative control siRNA; #p < 0.05, compared with NC.
(TIF)
