# Supplementary figures and images for: Sp1 Mediates a Therapeutic Role of MiR-7a/b in Angiotensin II-Induced Cardiac Fibrosis via Mechanism Involving the TGF-β and MAPKs Pathways in Cardiac Fibroblasts
Source: PLoS One. 2015 Apr 29;10(4):e0125513. doi: 10.1371/journal.pone.0125513 (PMC4414609; doi:10.1371/journal.pone.0125513)

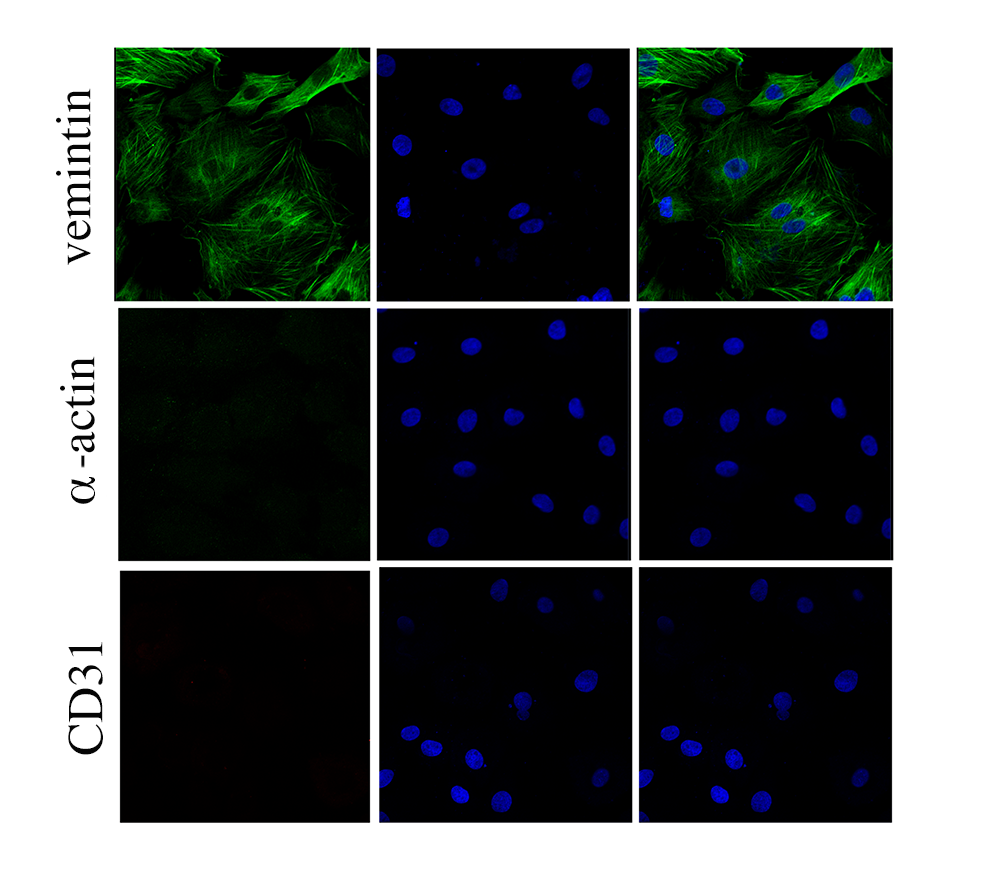

Supplement: S1 Fig — Immunofluorescent staining of vemintin (first row), α-actin (second row) and CD31 (third row) in CFs. (TIF) [file pone.0125513.s001.tif]

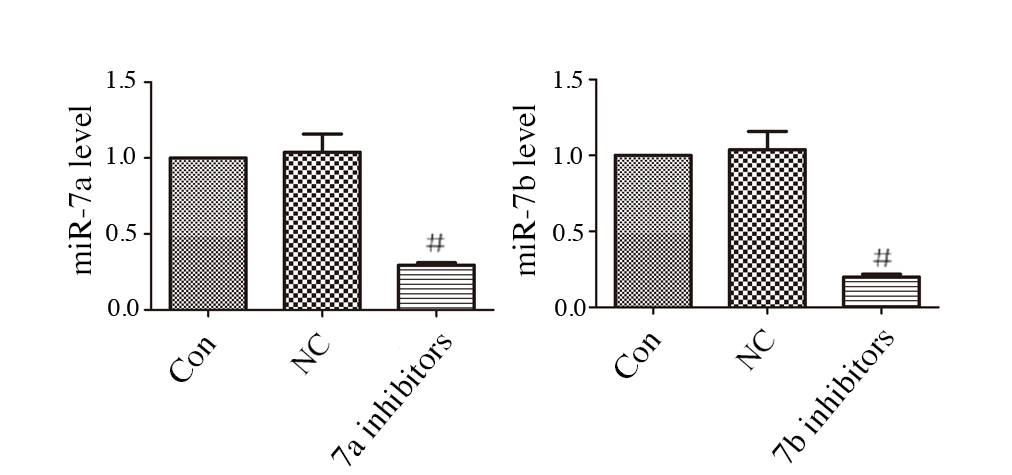

Supplement: S2 Fig — Con: normal untreated CFs; NC: negative control siRNA; #p < 0.05, compared with NC. (TIF) [file pone.0125513.s002.tif]
